# According, against, and above dietary norms: a key to understanding the relationship between personality style and taste preferences

**DOI:** 10.7717/peerj.8198

**Published:** 2019-12-06

**Authors:** Ligiana Mihaela Petre, Bianca Nicoleta Vatasescu

**Affiliations:** Department of Psychology/Faculty of Psychology and Educational Science, University of Bucharest, Bucharest, Romania

**Keywords:** Sensory liking, Taste preferences, Antisocial, Personality, Compulsive, Eating behavior, Personality traits, Sweet, Salt, Food

## Abstract

**Background:**

Understanding individual food preferences is critical for creating tailored strategies that promote healthy individual eating behaviors. Individual sensory liking appears to be an essential determinant of dietary intake. Taste preferences influence satisfaction and satiety, and may consequently influence weight status and psychological adjustment. The purpose of this study was to identify the association between taste preferences (sweet, salty, sweet & fatty, salty & fatty) and personality features.

**Methods:**

The Millon Clinical Multiaxial Inventory-III (MCMI-III) was used for the assessment of personality traits and PrefQuest (PQ) was used for measuring recalled food preferences. A total of 137 participants were included in the study. The relationship between compulsive and antisocial features and taste preferences was assessed by hierarchical multiple linear regression, while controlling for age, gender, BMI, marital status, and educational level.

**Results:**

The antisocial personality traits were a negative explanatory variable for sweet & fatty taste preference, *R*^2^ = .15, *t*(132) =  − 2.40, *p* = .018, 95% [−.57, −.06] and salty & fatty taste preference, *R*^2^ = .16, *t*(133) =  − 2.38, *p* = .019, 95% [−.07, −.01], while controlling for anthropological factors. In addition, men showed a higher preference than women for sweet & fatty food, such as chocolate or desserts, *r*_*sp*_ = .19, *p* = .021, and for the salty & fatty food, *r*_*sp*_ = .30, *p* < .001. BMI was not found to moderate the relationship between personality and taste preference. No significant association was found between compulsive personality traits and food preference, as assessed by sensory liking.

**Conclusions:**

The findings can bring a much better understanding of the relationship between the compulsive or antisocial personality and taste preferences. In addition, it may help build psychotherapeutic and nutritional strategies that promote healthy eating behaviors, tailored to a particular personality style.

## Introduction

Taste preference is among many factors that have been linked to the development of obesity (Aguayo et al., 2012). Governments and health organizations have developed dietary guidelines for promoting healthy lifestyles and for reducing salt, sugar, and fat intake (World Health Organization, 2012; World Health Organization, 2013). However, individual personality traits and eating styles may influence the probability of respecting these recommendations. Hence, personality style may be a decisive factor in choosing an unbalanced diet.

Understanding the link between taste preferences and personality can contribute to the prevention of lifestyle-related diseases because high intake of fat, salt, and sugar can increase the risk of non-communicable diseases (WHO, 2015). Obesity is also associated with impulsivity (Thanos et al., 2015) and the consumption of highly palatable and energy-dense foods, rich in fat and sugar. Suboptimal diet is responsible for more deaths than any other risks, globally, including smoking (Forouzanfar et al., 2015; Vos et al., 2017). Therefore, the urgent need to improve human diet is highlighted in recent publications.

Previous data suggest that personality traits may influence eating styles (Heaven et al., 2001) and food choices (Mõttus et al., 2013; Mõttus et al., 2012; Tiainen et al., 2013). People will experience more pleasure, satisfaction, and satiety when eating the food they like instead of the one they like less (Mattes & Vickers, 2018).

### Liking, wanting, and preference

Reward components, known in the literature as liking and wanting, have a high impact on human appetite behavior (Finlayson & Dalton, 2012) and overeating (Pool et al., 2016). Wanting refers to the motivation to obtain a reward and liking is the hedonic pleasure felt during its consumption (Robinson & Berridge, 2001).

We use the concepts of “liking”, “wanting”, and “preference” in this article. As “wanting” and “liking” are related to subjective rewards and are widely used to refer to addictions, the term “preference” is used to name the option for food at the expense of other relevant alternatives at the time of choice (Frewer & Van Trijp, 2007). The term “liking” is the evaluation of quality, the emotional acceptance of the specific food, and the acceptance of the experience of pleasure associated or not, with the product itself. On the contrary, the term “preference” is used to express a choice, more precisely an indication of two or more alternatives presented together, bearing in mind that at any given time and context, some specific options are more desirable than others (Franchi, 2012).

### Tastes

Previous data show that taste has an essential influence on food choices (Honkanen & Frewer, 2009; Kourouniotis et al., 2016). The sense of taste, one of the five primary senses, is innately hedonic and biased. The categories of taste—sweet, sour, salty, bitter and umami—are linked to evolution: the identification and ingestion of nutrients and the avoidance of poisons. It is known that sweetness provides both energy and essential nutrients for humans. Moreover, sugars have properties that reduce pain and have been reported to alleviate depression, premenstrual symptoms, or responses to stress (Drewnowski et al., 2012). The perception of sweetness suppresses the oral perception of fat. Sweet & fat comfort foods are perceived as sweet and not fat, as the sucrose creates a perceptual illusion. Despite worldwide initiatives to reduce sodium intake, salt improves the sensory properties of food. Sodium chloride imparts an almost pure salty taste, whereas potassium chloride tastes both salty and bitter. Salt was found to increase the perception of a product’s thickness, intensify sweetness, mask chemical notes, and improve overall flavor intensity. Moreover, sodium may suppress bitter tastes. The suppression of bitter compounds may improve the taste attributes of other food components. In conclusion, adding sodium to mixtures between sugar and bitter enhanced the perceived sweetness of the mixture because of sodium suppressing bitterness and releasing sweetness (Breslin & Beauchamp, 1997). Therefore, salt plays a role in enhancing the palatability of food’s flavor, beyond imparting a desirable, salty taste. Positive “liking” versus negative “disgust” expressions can be seen on the first postnatal day. Sweet tastes elicit positive hedonic “liking” expressions comprising relaxed facial muscles and a contented licking of the lips, whereas bitter tastes elicit “disgust” expressions (Berridge & Kringelbach, 2015). Understanding the interactions between the categories of tastes plays a vital role in the development of food choices.

### Taste preference and personality traits

Sensation seeking is one of the personality traits that have been studied in association with food choices. Higher scores in sensation-seeking are associated with an increased preference for spicy food (Byrnes & Hayes, 2013; Ludy & Mattes, 2012) and caffeine (Mattes, 1994). To our knowledge, sweet taste preference is the most studied taste, so far. Higher levels of agreeableness and neuroticism (Keller & Siegrist, 2015; Kikuchi & Watanabe, 2000) are linked to sweet taste preference. Preference for sweet white wine over dry white wine is associated with more trait neuroticism and lower levels of openness (Saliba, Wragg & Richardson, 2009). However, the association between sweet taste preference and personality traits is contradictory. Sweet taste preference has been linked to depression in experimental studies, while there are other studies, which claim that depressive persons do not search for pleasure and, therefore, correlate negatively with sweet taste preference (Scinska et al., 2004).

Spicy food and aggression are related (Sagioglou & Greitemeyer, 2016). Bitter taste preferences were associated with antisocial personality traits. Words such as “hot” images (e.g., the color red, which is often the color used to depict violence or blood) and physical sensations (e.g., increase in body temperature) are associated with both spicy food and aggressive intent. Using words associated with hot temperature has been found to increase aggressive thoughts and hostile intentions in participants (Nathan DeWall & Bushman, 2009). Moreover, spicy foods contain capsaicin, an ingredient that evokes discomfort, irritation, and even pain (Bègue et al., 2015). It is known that discomfort and pain can evoke aggression. As a result, the aversive physiological reactions evoked by consuming spicy food can elicit aggressive behavior (Batra, Ghoshal & Raghunathan, 2017).

Neuroticism is associated with sweet and savory food consumption. Neuroticism causes emotional and external eating and indirectly leads to sweet and savory food eating. As might be expected, people that score high in neuroticism eat more high-energy dense sweet and savory food and seem to adopt counter-regulatory emotional eating. On the other hand, conscientiousness is negatively correlated with sweet and savory food. People who score high in conscientiousness may adopt regulatory restrained eating, consume more fruits and vegetables, and consume less sweet and savory food, meat, and sweetened drinks (Meier et al., 2012). Meier et al. (2012) reported that sweet taste preferences were positively linked to prosocial personality characteristics. Individual preference for sweet foods predicted prosocial personalities, prosocial intentions, and prosocial behaviors. Moreover, people tend to associate agreeability with sweet food consumption. As shown, people indicated strangers that liked sweet foods, such as candy, as having higher scores on agreeableness. Participants’ self-reports of agreeableness and helping behavior increased after eating sweet food, compared to eating a non-sweet food (Meier et al., 2012).

However, the number of studies investigating the association between taste preference and personality is limited. The theoretical basis for personalized nutrition is underdeveloped (Ordovas et al., 2018). Personalized dietitian recommendations based on the history of individual and food preferences are important for the development of healthy eating behaviors in the future. In order to create tailored eating strategies, it is necessary to first understand the association between food preference and personality. Measuring the preference of the corresponding sensory likings in association with personality traits contributes to understanding the determinants of dietary behaviors.

Moreover, personalized diets and interventions are known to be more effective than population-based guidelines. It has become clear that there is a considerable inter-individual variation in response to dietary interventions, and some interventions may help certain individuals or population subgroups more than others, depending on their genotype, phenotype, and environment (De Roos & Brennan, 2017).

The present study has the overall goal to increase our understanding of the associations between taste preference and personality traits.

## Material & Methods

The participants were 157 participants, 79 men, and 78 women. The sample characteristics regarding gender, age, education, place of residence, marital status, any individual eating plans or diets, and level of body mass index (BMI), are shown in Table 1.

**Table 1:** Baseline characteristics (*N* = 157).

**General characteristics**		**Frequency**	**Percent**	
Gender	Male	79	50.3	
	Female	78	49.7	
Marital status	Single	103	65.6	
	Married	38	24.2	
	Divorced	5	3.2	
	Missing	11	7.0	
Residence	Urban	125	79.6	
	Rural	20	12.7	
	Total	145	92.4	
	Missing	12	7.6	
Education level	Middle school	17	10.8	
	High school	29	18.5	
	University	69	43.9	
	Postgraduate	39	24.8	
	Missing	3	1.9	
Dieting	Dieting	45	28.7	
	No dieting	110	70.1	
	Missing	2	1.3	
Age (years), cut-points	≤22.00	51	32.5	
	23.00–24.00	30	19.1	
	25.00–38.00	39	24.8	
	39+	37	23.6	
	Total	157	100.0	
BMI (kg/m^2^), cut-points	≤20.31	39	24.8	
	20.32–22.72	38	24.2	
	22.73–26.17	39	24.8	
	26.18+	38	24.2	
	Missing	3	1.9	
	Mean	Median	Std. Deviation	Missing
Age, years	30.52	24.00	12.67	0
BMI, Kg/m^2^	23.09	22.55	4.35	3

### Instruments

### Taste preferences

The preference for salty, sweet, sweet & fatty, and salty & fatty, was assessed with “PrefQuest” (Deglaire et al., 2012), which measures recalled liking for the four sensations: salty, sweet, fatty and salty, and fatty and sweet. PrefQuest (PQ) includes four types of items: sweet, fatty & sweet, salty, and salty & fatty preferences. These refer to: the level of seasoning, by adding salt, sweeteners or fat; preferences for types of dishes in a restaurant menu; overall questions about sweet-, salty-, and fat-related behaviors (Deglaire et al., 2015; Deglaire et al., 2012; Lampuré et al., 2016; Lampuré et al., 2014). PrefQuest (PQ) was designed to measure the relationships between nutrition and health (Deglaire et al., 2012). In the initial report, PQ was administered through the web-based “Nutrinet Santé Study” and was filled in by 47,803 participants, of whom 77% were women and 23% were men. Participants described PQ as short, easy and entertaining. The completion time for PQ items lasted, on average, 23.5 min (Deglaire et al., 2012). PQ is the first internally validated questionnaire, which proposes a liking score to be calculated based on various types of items, that include liking for foods, preferred seasoning level, and a few items related to dietary behaviors. The factors of each scale had good psychometric properties. All items exhibited a rather good repeatability, with an average intra-class correlation coefficient (ICC) higher than 0.7. The underlying structure within each of the four sensations (sweet, fatty-and-sweet, fatty-and-salty, and salty) was determined by exploratory factor analysis, and then internally validated by confirmatory factor analysis. Factorial analyses were performed on the validity study. There were ratios of 4–6 for the sweet, fatty-and-sweet, and fatty-and-salty scales (Deglaire et al., 2012). Scales exhibited a theoretically good factor structure, being unidimensional for the salty scale and with interrelated sub-dimensions for the sweet, fatty-and-salty, and fatty-and-sweet scales. For each factor, internal consistency, convergent and divergent validities were demonstrated (Deglaire et al., 2012). Positive correlations between PQ and sensory test measurements in the laboratory have been shown. Therefore, PrefQuest has proven to be valid, repeatable, feasible, and can thus serve as a proxy for sensory test measurements of liking (Lampuré et al., 2014).

### Personality traits

Millon Clinical Multiaxial Inventory-III (MCMI-III) was used to identify and measure personality traits. MCMI-III is a 175-item, true-false self-report measure. The inventory contains 24 clinical scales arranged into four distinct categories: Clinical Personality Patterns, Severe Personality Pathology, Clinical Syndromes, and Severe Clinical Syndromes. MCMI personality disorder (PD) scales have exhibited good levels of internal consistency throughout the years, although two MCMI–III measures (Compulsive and Narcissistic scales) exhibited less than desirable values (coefficient *α* = .66 and .67). Retest intervals between 5 days and 4 months have provided a median value of reliability, across the personality disorder (PD) scales of *r* = .78, ranging from .58 (Depressive scale) to .93 (Strack & Millon, 2007). MCMI-III has three stages of validation, is closely aligned with the DSM-IV classification system, and is associated with Theodore Millon’s comprehensive evolutionary theory (Jankowski, 2004; Pincus & Krueger, 2015).

### Procedure

The non-proportional stratified sampling methods were used on the population within different clinical settings. Then, from each stratum, a simple random sample was selected. The strata cover two hospitals (*N*: 2 × 100), one probation office (*N*: 1 × 50), one Institute of Psychological services (*N*: 1 × 50), two psychiatrist private practices (*N*: 2 × 25), six psychological private practices (6 × 25), four general practitioner private practices (*N*: 4 × 25). A number of 687 persons participated in the study. From those participants, 157 participants were selected. Inclusion criteria referred to antisocial and compulsive personality features, established by a psychological evaluation using the Multiaxial Clinical Millon Inventory—III (MCMI-III) assessment. The lifetime absence of psychiatric illness was established through a clinical interview. Exclusion criteria were comprised of: a diagnosis of severe personality disorders or other neurological/medical conditions, known to affect mental health.

The participants were informed about the purpose of the study to explore the association between personality traits and taste preferences. Each participant was evaluated separately within a clinical setting. The participants were invited to a psychological assessment, starting with a personality assessment. The instructions were presented in the same manner for every participant. A printed version of MCMI-III and, thereafter, one of PQ were provided to each participant. The participants were asked to complete the items of PQ according to their general taste preferences irrespective of their current diet or eating behavior.

Research Ethics Committee of the University of Bucharest approved the current study (IRB no. 03/21.01.2019). The procedure complied to the ethical standards of the College of Psychologists from Romania and the American Psychological Association. Participants gave their written informed consent, and pseudonyms were used to protect their anonymity.

### Statistical methods

Data analysis was performed in several steps. Descriptive statistics were calculated for study variables. The cases with missing data were deleted. The cut-points were created for age and BMI, using visual binning. The data has been divided into even percentiles of a number of cases, each bin containing the same number of cases. The data that indicated extreme values was mapped, using boxplots, and was manually changed with the largest value that was not considered an outlier. In addition, the data was checked for the assumption of normality, accuracy and the presence of outliers, using Skewness and Kurtosis. The skewed data was treated by square root transformation or log 10 transformation. Moreover, the linearity, as one of the assumptions for multiple linear regression, were examined by plotting scatterplots of the relationship between each explanatory variable and the outcome variable. Durbin-Watson test was used for checking the presence of autocorrelations in residuals. The Mahalanobis Distance, Covariance ratio, Cook’s distance, and centered leverages were calculated for detecting unusual and influential data. Adding to this, P–P plots of standardized residuals against the predicted values assessed linearity and homoscedasticity of the residuals. The absence of multicollinearity was checked, using the variance inflation factor (VIF) and tolerance. Therefore, we conducted bivariate and partial correlations between continuous variables, using Pearson’s correlation. The categorial variables with more than two categories (marital status and educational level) were recoded into dummy variables. Three dummy variables of marital status (single, married and divorced) and four dummy variables of educational level (middle school, high school, university and postgraduate degree) resulted. In addition, the associations between nominal and interval scaled variables were calculated using univariate Anova analyses and Eta-squared statistics.

Exploratory analysis has highlighted the potential associations between the respective variables. The structural models were created, according to the literature on factors of taste preferences and personality, and to the results of correlations between variables.

Furthermore, we conducted a hierarchical multiple linear regression, to ascertain the extent to which personality traits predict taste preferences. In the hierarchical regression analyses, personality traits were treated as an explanatory variable for taste preferences. Hence, although we use personality traits as the independent variable, we do not suggest that this is the only pathway of influence. During the first steps of regression analyses we introduced the controlling variables, which were found to have effects on the dependent variable. In the first block age and gender were added. In the second block, BMI was entered, if it added a significant contribution to the model, after controlling for age and gender. During the third step, marital status (single, married) and/or education level (middle school, high school, university) were added. The personality variable was entered in the final step, to analyze its contribution, after controlling for the previously entered predictors. For the second, third, fourth and/or fifth block, the forward method was applied to obtain the simplest model. The criteria for including a variable was the significance of the regression coefficient, at a *p* < .01 level. This hierarchical regression approach enabled the investigation of whether gender, BMI, marital status or educational level enhanced the prediction model and whether personality, introduced at the last block, had a significant contribution to the previous model.

For all statistical analyses, the Statistical Package for Social Sciences (SPSS) was used.

## Results

### Descriptive statistics

The participants were 157 subjects (*M* = 30.5, SD = 12.7), 79 men and 78 women, with an age range of 18–80 years. Twenty cases have had missing values and they were removed. Screening for outliers, using box plots revealed single construct outliers, which had data values that were unusually large or small compared to the other values of the same construct. The boxplot analyses showed three BMI outliers (cases 14, 19, 16) and four age outliers (44, 50, 105, 106). All outliers were cases with extreme values, which were manually changed with the largest value that was not considered an outlier. The final number of cases remained *N* = 137; 74 men (*M* = 29.3, SD = 12.6) and 63 women (*M* = 31.7, SD = 12.4). Age was non-normally distributed, with skewness of 1.25 (SE = .21) and kurtosis of .30 (SE = .41). A square root transformation did not solve the positive skewness (1.07, SE = .21). Log 10 transformation conducted to more symmetric data of age distribution (skewness 89, SE = .21). Table 2 shows the characteristics of the sample included in the statistical analyses (frequencies and percentages for categorical variables, as well as means, median, standard deviation, and interquartile ranges for continuous variables).

**Table 2:** Baseline characteristics (*N* = 137).

**General characteristics**		**Frequency**	**Percent**	
Gender	Male	74	54	
	Female	63	46	
Marital status	Single	98	71.5	
	Married	35	25.5	
	Divorced	4	2.9	
Residence	Urban	118	86.1	
	Rural	19	13.9	
Education level	Middle school	14	10.2	
	High school	27	19.7	
	University	65	47.4	
	Postgraduate	31	22.6	
Dieting	Dieting	42	30.7	
	No dieting	95	69.3	
Age (years), cut-points	18.00–21.00	36	26.3	
	22.00–24.00	38	27.7	
	25.00–38.00	30	21.9	
	39.00–61.00	33	24.1	
	Total	137	100.0	
BMI (kg/m^2^), cut-points	16.73–20.32	35	25.5	
	20.33–22.86	35	25.5	
	22.87–26.26	33	24.1	
	26.27–34.60	38	24.8	
	Total	137	100.0	
	Mean	Median	Std. Deviation	IQR
Age, years	30.26	24.00	12.54	17
BMI, Kg/m^2^	23.64	22.86	4.19	5.96

### Taste preferences and antisocial personality traits

The results showed a statistically significant relationship between antisocial features and fatty & sweet food preferences (P1 sweet & fatty) scores, Pearson’s *r*(135) =  − .18, *p* = .037. Another statistically significant association was between antisocial traits and salty & fatty taste preferences (P4 salty & fatty), Pearson’s *r*(135) =  − .18, *p* = .035. Moreover, the results of two-tailed partial correlation between antisocial traits and sweet & fatty preference (P1 sweet & fat), *pr*(134) =  − .20, *p* = .021, and the salty & fatty dietary behavior (P4 salty & fatty), *pr*(134) =  − .19, *p* = .024, while controlling for age, were negatively statistically significant. The results of two-tailed partial correlation between antisocial personality and taste preference, while controlling for BMI, were statistically non-significant.

Analyses of variance showed a main effect on sweet & fatty taste preference (P1 sweet & fatty) of the following variables: male as gender, *F*(1, 135) = 5.33, *p* = .022, ${\eta }_{p}^{2}=.038$; single marital status, *F*(1, 135) = 5.34, *p* = .038, ${\eta }_{p}^{2}=.038$; married marital status, *F*(1, 135) = 4.40, *p* = .013, ${\eta }_{p}^{2}=.045$; middle school, *F*(1, 135) = 5.42, *p* = .021, ${\eta }_{p}^{2}=.039$, and university as educational levels *F*(1, 135) = 6.32, *p* = .013, ${\eta }_{p}^{2}=.045$. In addition, the variance in the salty & fatty taste preference (P4 salty & fatty) was explained by the gender *F*(1, 135) = 15.36, *p* < .001, ${\eta }_{p}^{2}=.102$, single *F*(1, 135) = 4.00, *p* = .048, ${\eta }_{p}^{2}=.029$ and married status *F*(1, 135) = 4.08, *p* = .045, ${\eta }_{p}^{2}=.029$. The main effects of gender (male) and marital status (single or married) on salty & fatty taste preference were qualified by the following interactions: male and single status *F*(1, 131) = 6.05, *p* = .015, ${\eta }_{p}^{2}=.044$; male and married *F*(1, 131) = 4.96, *p* = .028, ${\eta }_{p}^{2}=.036$. All other main effects and interactions were non-significant, all *F* ≤ .00, *p* ≥ .993, ${\eta }_{p}^{2}\leq .001$.

### Sweet & fatty taste preference and antisocial personality traits

Regression diagnostics showed that the assumption of independence and collinearity were met (Durbin-Watson statistic = 2.19, antisocial, VIF = 1.00, tolerance = .99, age, VIF = .90, tolerance = 1.11, males, VIF = .96, tolerance = 1.05, middle school education, VIF = .91, tolerance = 1.10). Minimum and maximum values of the standard residuals were between −2.83 and 2.76. The covariance ratio maximum and minimum for the model was CVR_i_ = [.80, 1.20]. Thus, all cases were between the CVR_i_ interval limits. The average leverage was .029. One case had values greater than three times of average leverage value, *h*_9_ = .11. The results of Mahalanobis Distance test did not show any influential cases. Moreover, all cases were found to have proper Cook’s Distances. P–P plots of standardized residuals against the predicted values assessed linearity and homoscedasticity, showing some deviation from normality between the observed cumulative probabilities of 0.3–0.4, and 0.6–0.8, but it appears to be minor.

The association between sweet & fatty taste preference and antisocial features was analyzed with hierarchical multiple regression, in five steps. First, age and gender were entered. Second, BMI was added, to evaluate if it contributed significantly, after controlling for age and gender. In the next step, two dummy variables of marital status (single and married) were added. In the fourth step, three dummy variables of educational level (middle school, high school and university education) were added. The personality variable was added in the last step, to investigate whether antisocial personality variable enhanced the previous model, after controlling for the previously-added variables.

The results of the hierarchical multiple regression revealed that at step 1, age and male gender contributed significantly to the regression model, *F*(2, 134) = 5.93, *p* = .003, and accounted for 8.1% of the variation of sweet & fatty taste preference (P1 sweet & fatty). BMI, marital status, and university variables were not found to have a significant contribution to the regression model. Introducing the two dummy variables of educational level (1 = middle school; 0 = other) explained an additional 2.9% of the variation of sweet & fatty taste preference. This change in R was statistically significant, *F*(3, 133) = 5.48, *p* = .001. Adding the antisocial personality variable in the regression model increases *R*^2^ by .04, making the *R*^2^ = .15, *F*(4, 132) = 5.70, *p* < .001. The final model explained an additional 3.7% of the variation in sweet & fatty taste preference. Overall, the final model explained 15% of the sweet & fatty taste preference scores. Based on the *β* coefficients, the results showed that antisocial traits, *β* =  − .20, *t*(132) =  − 2.40, *p* = .018, 95% [−.57, −.06], and male gender, *β* = .20, *t*(132) = 2.34, 95% [2.31, 27.55], *p* = .021, had the most significant contribution to the final model. In addition, sweet & fatty preference (P1 sweet & fatty) was negatively related with middle education, *β* =  − .18, *t*(132) =  − 2.16, *p* = .032, 95% [−44.60, −1.99], and age, *β* =  − .17, *t*(132) =  − 2.03, *p* = 046, 95% [−83.99, −1.05].

In the final model (*R*^2^ = .15, *p* < .001), approximately 4% of males had a higher preference than women for the sweet & fatty food, such as chocolate or desserts, *r*_*sp*_ = .19, *p* = .021. In addition, 4% of people with low antisocial personality scores liked sweet & fatty food, such as desserts, *r*_*sp*_ =  − .20, *p* = .018. Moreover, young age explained sweet & fatty taste preference, *r*_*sp*_ =  − .16, *p* = .045.

### Salty & fatty taste preferences and antisocial features

Regression diagnostics showed that multicollinearity was not found in the explanatory variables (antisocial, VIF = 1.00, tolerance = .99; gender, VIF = 1.02, tolerance = .98). Residuals met the assumption of independence (Durbin–Watson = 1.79). Minimum and maximum values of the standard residuals were between −2.14 and 2.68. The covariance ratio maximum and minimum for the model was CVR_i_ = [.87, 1.13]. Thus, all cases were between the CVR_i_ interval limits. The average leverage was .022. All cases had proper average leverage, Mahalanobis Distance, and Cook’s Distances values. The scatterplot of the standardized residuals against standardized predicted values suggests the presence of linearity and homoscedasticity, because the points are mostly randomly and evenly dispersed through the plot. In the P–P plot, the dots lie almost exactly along the diagonal. Overall, it does not appear to be a severe problem with non-normality of residuals in the model.

The association between salty & fatty taste preference and antisocial features were analyzed with hierarchical multiple regression, in four steps. First, gender and age were entered. Second, BMI was added to evaluate its contribution to the model, after controlling for age and gender. In the next step, two dummy variables of marital status (single and married) were entered. The personality variable was added in the last step, to investigate whether antisocial personality variable enhanced the previous model, after controlling for the previously-added variables.

The results of the hierarchical multiple regression revealed that at step 1, age and male gender contributed significantly to the regression model, *F*(2, 134) = 9.14, *p* < .001, and accounted for 12% of the variation of salty & fatty taste preference (P4 salty & fatty). BMI, single and married status, were not found to have a significant contribution to the model. Adding antisocial variable to the regression model increases *R*^2^ by .04, making the *R*^2^ = .16, *F*(3, 133) = 8.19, *p* < .001. Based on the *β* coefficients, the results showed that males, *β* = .30, *p* < .001, *t*(133) = 3.72, 95% [1.34, 4.38] had the most significant contribution to explain the salty & fatty preference scores, followed by antisocial traits, *β* =  − .19, *p* = .019, *t*(133) =  − 2.38, 95% [−.07, −.01]. Age did not add any statistically significant contribution in the final model (*β* =  − .15, *p* = .070). In the final model (*R*^2^ = .16, *p* < .001), approximately 9% of males showed a higher preference for a salty & fatty diet than women, *r*_*sp*_ = .30, *p* < .001. In addition, antisocial personality uniquely explained 4% of the variation of salty & fatty taste preference, *r*_*sp*_ =  − .19, *p* = .019.

### Taste preferences and compulsive personality traits

The results do not show any statistically significant associations between compulsive personality and taste preferences, including one-tailed or two-tailed partial correlations, while controlling for anthropometric factors.

## Discussion

In this study, the association between taste preferences and antisocial personality traits were investigated on 137 participating adults. Taste preferences, which are a strong determinant of dietary intake and weight status, interact with other determinants of dietary behavior (Lampuré et al., 2015). Physiological factors, such as age and gender, are associated with dietary intake (Fraser et al., 2000; Hawkes et al., 2015; Lampuré et al., 2015). Our results show that there is a statistically significant relationship between gender and food selection. 9% of the variance of salty & fatty dietary behavior and 4% of the variance of sweet & fatty taste preference was explained by male gender. There was a statistically significant male preference to add butter or mayonnaise for more saltiness and fattiness, or to select dessert, as a preferred food. In addition, our results suggest that there is a negative significant association between young men with middle school education, and the preference for chocolate, pastries or other desserts. In accordance with previous results regarding the association between individual characteristics such as psychological, socio-demographic, economic and lifestyle factors and dietary intake (Méjean et al., 2011) and weight status (Godley & McLaren, 2010; Lampuré et al., 2015), our results show that there is a statistically significant interaction effect between male gender and marital status (single or married), which explains the salty & fatty taste preference. Single or married men reported a specific salty & fatty behavior. Furthermore, the results of previous research on the relationship between taste preferences and BMI are contradictory. It is known that eating behaviors such as cognitive restraint, uncontrolled eating, and emotional eating are strongly related to unhealthy intake and BMI (Lampuré et al., 2015). Regarding the relationship between BMI and food liking, the results of a recent literature review (Wall et al., 2019) suggest a positive association (33%), a negative one (11%), or no association (56%) between food liking and BMI. In this light, our study results show no significant influence of BMI on preference for desserts or on salty & fatty dietary behavior. BMI was not shown to contribute to the hierarchical model of the relationship between antisocial traits and sweet & fatty taste preference, or salty & fatty dietary behavior.

It is known that psychological factors influence dietary behaviors. Building on previous research, which showed that sweet taste preference was significantly associated with neurotic personality traits and that a strong sweet taste preference was linked to personality traits in obesity (Elfhag & Erlanson-Albertsson, 2006), our findings point that there is a relationship between antisocial personality traits and taste preferences. The conflicting personality, as in the case of the antisocial personality, was described as being more outgoing and implies being more unconcerned and carefree (Hoffmann et al., 2019; Millon, 2011). In spite of the previously reported tendency of the conflicting personality to prefer more intense sweetness, our results showed that the sweet & fatty food selection is negatively associated with antisocial features. It is known that the hedonic reward of sweet and fatty foods triggers a drive for eating, and impulsive choice has been associated with a preference for concentrated solutions, which are high in sweetness (Weafer, Burkhardt & De Wit, 2014).

The antisocial personality has a high level of impulsivity, which is sustained by the tendency to engage in impulsive actions, but our findings show that antisocial features are a negative explanatory variable for sweet & fatty food selection, while controlling for age, gender, BMI, marital status, and educational level. We also found that young men with lower levels of antisocial features like, more than women, the sweet & fat food, as desserts.

The antisocial personality is positively linked to spicy and bitter foods (Sagioglou & Greitemeyer, 2016). As salt suppresses bitter compounds, we expected a negative relationship between antisocial personality traits and salty taste preference. In this light, our results show that antisocial personality features are a negative explanatory variable for the salty & fatty taste preference, while controlling for age, gender, BMI, marital status (single and married), and educational level (middle school, high school, university, and postgraduate degree). Our data, along with those obtained by Sagioglou & Greitemeyer (2016), could indirectly confirm the dietary behavior of the antisocial personality, regarding the preference of spicy and bitter taste, based on the negative association with salty & fatty taste preference.

If the antisocial behavior was described by the tendency to act against or above the common norms (Millon, 2011), the current study results show that people with antisocial traits report a normative dietary behavior, by complying to the dietary guidelines for promoting healthy lifestyles and for reducing salt, sugar, and fat intake. The antisocial traits are negative predictors for chocolate and other dessert’s preferences. Moreover, antisocial features are negative significant predictors for the saltiness and fattiness dietary behavior, such adding butter or salty & fatty sauces to foods. Our results show that antisocial traits in young men are associated with complying to dietary norms, by conforming to the actual dietary norms of reducing sugar and sodium intake. On the other hand, the compulsive personality, which has been described in the scientific literature as being more conforming with the general societal rules (Millon, 2011), was not statistically significantly associated with any dietary norms or taste preferences. We cannot state that compulsive personality features are not complying with the dietary norms; the statistical results did not highlight any significant trend of the eating behaviors of this studied population. In this light, the results of our study could be an appropriate key to a better understanding of the relationship between antisocial and compulsive personality and taste preferences.

One of the limits of the present study is the assessment of only four tastes. Secondly, the taste preference was measured by recalled liking. Another limitation was the use of a small number of covariates, to assess the potential lifestyle and health characteristics of the participants, including the level of hunger. Future studies should consider a larger sample, and an experimental study, in order to better control taste preferences, and include bitter, spicy, and umami tastes, or multiple tests to assess recalled liking.

## Conclusions

The statistically significant results suggest that antisocial personality traits are a negative predictor for sweet & fatty and salty & fatty taste preferences, while controlling for age, gender, BMI, marital status and educational level. Adding to this, young men showed a higher preference than women for sweet & fatty foods, such as chocolate or desserts and for salty & fatty foods. BMI factors were not found to influence the relationship between personality and taste preference. No significant association was found between compulsive personality traits and food preference, as assessed by sensory liking. The results show that future psychological and nutritional interventions must be tailored to personality, taste preferences and eating behaviors. The findings can contribute to an increased understanding of the link between compulsive personality, antisocial personality, and food preferences, and may help build psychotherapeutic and nutritional strategies, for promoting personality-based healthy eating behaviors, thus preventing the onset of obesity and eating disorders.

##  Supplemental Information

10.7717/peerj.8198/supp-1 Supplemental Information 1 Personality style and taste preferences datasets

## Additional Information and Declarations

### Data Availability

The following information was supplied regarding data availability: Raw data is available as Dataset S1.
